# Unlocking the Future: New Biologic Therapies for Rheumatoid Arthritis

**DOI:** 10.7759/cureus.72486

**Published:** 2024-10-27

**Authors:** Sarika J Patil, Vandana M Thorat, Akshada A Koparde, Somnath D Bhinge, Dhanashri D Chavan, Rohit R Bhosale

**Affiliations:** 1 Department of Pharmacology, Krishna Institute of Medical Sciences, Krishna Vishwa Vidyapeeth (Deemed to be University), Karad, IND; 2 Department of Pharmaceutical Chemistry, Krishna Institute of Pharmacy, Krishna Vishwa Vidyapeeth (Deemed to be University), Karad, IND; 3 Department of Pharmaceutical Chemistry, Rajarambapu College of Pharmacy, Kasegaon, IND; 4 Department of Pharmaceutics, Krishna Foundation’s Jaywant Institute of Pharmacy, Wathar, IND

**Keywords:** biologic therapy, disease-modifying antirheumatic drugs (dmards), gene therapy, interleukin blockers, rheumatoid arthritis (ra), tnf inhibitors

## Abstract

Rheumatoid arthritis (RA) is a chronic autoimmune disorder that leads to joint destruction and functional disability. Traditional treatments, including disease-modifying antirheumatic drugs (DMARDs), often fail, leaving many patients without remission. The advent of biologic therapies that target specific immune system components (e.g., cytokines, T cells) has transformed RA treatment by offering new management options. These biologics (e.g., TNF inhibitors, interleukin blockers) are highly effective in controlling disease activity and preventing joint destruction. However, their use comes with safety concerns, particularly regarding immunosuppression and infection risks. Although still experimental, studies predict that future research will focus on enhancing the clinical response and safety of these agents through personalized approaches or novel mechanisms of action.

## Introduction and background

Rheumatoid arthritis (RA) is a chronic inflammatory disease that causes significant morbidity and disability. It primarily affects the joints but can also involve other parts of the body. Common symptoms include joint swelling, morning stiffness, and fatigue. Although the exact cause of RA is unknown, genetic susceptibility has been identified. Researchers are investigating environmental factors, such as certain oral bacteria and drug exposures, that may trigger the disease. The activation of the immune system may lead to the breakdown of tolerance to T cell and B cell epitopes, resulting in the invasion of autoantibodies and inflammatory cells into the joints. This process promotes synovial hyperplasia and the proliferation of tissue-destroying macrophages and fibroblasts, leading to bone and cartilage erosion within the joint capsule and causing severe physical impairment. RA complications can include cardiopulmonary issues, and early treatment aimed at achieving remission is believed to be crucial for improving long-term outcomes [1-5]. RA affects about 0.5%-1% of the global population, typically occurring in individuals aged 30 to 60, with women being three times more likely to develop the disease than men. Patients often seek medical help due to joint pain, stiffness, fatigue, loss of appetite, muscle soreness, or fever. Symptoms usually worsen gradually over weeks to months, but some patients experience sporadic "flare-ups" with intensified symptoms followed by periods of relief [6,7]. RA is marked by symmetric joint inflammation and abnormal immune responses to autoantigens. Early in the disease, the production of anti-citrullinated protein antibodies (ACPA) occurs, and both B and T cells become activated in the synovium. This activation leads to the thickening of the synovial membrane and hyperplasia of the lining layer, along with the infiltration of immune cells. Plasma cells migrate into germinal centers in the tissue, where they undergo affinity maturation. B cells and T cells become effector cells, further infiltrating the tissue and promoting autoimmunity. Macrophages exhibit inflammatory properties, and synovial fibroblasts express destructive marker proteins. The aggregation of these cells leads to invasive growth and the destruction of cartilage and bone [8,9].

## Review

Pathophysiology

Traditional treatments for RA include disease-modifying antirheumatic drugs (DMARDs) such as methotrexate, minocycline, gold, leflunomide, sulfasalazine, and hydroxychloroquine. However, only about 30% of patients achieve remission, and 20% do not respond at all. The effectiveness of these drugs is influenced by genetic and environmental factors. New treatment regimens with non-biological DMARDs and combinations like triple therapy have been developed, but few can provide long-term remission. RA is characterized by chronic synovial proliferation and inflammation that affects cartilage and bone. The exact mechanisms are unclear, but genetic, hormonal, and environmental influences are strongly implicated [10-12]. Genetic risk factors include the major histocompatibility complex (MHC) class II allele DR4, which increases RA risk. Transgenic mice with human DR4 develop a disease similar to RA. Environmental factors like smoking, silica dust exposure, and viral infections (e.g., parvovirus B19, HIV, HTLV-1, Epstein-Barr virus) are also involved in RA development. These viruses may induce polyclonal B cell activation. Hormones, especially estrogens, may play a role in gender differences in RA incidence, interacting with genetic risks to influence disease pathogenesis [13-15]. 

Current treatment landscape

**Figure 1:**
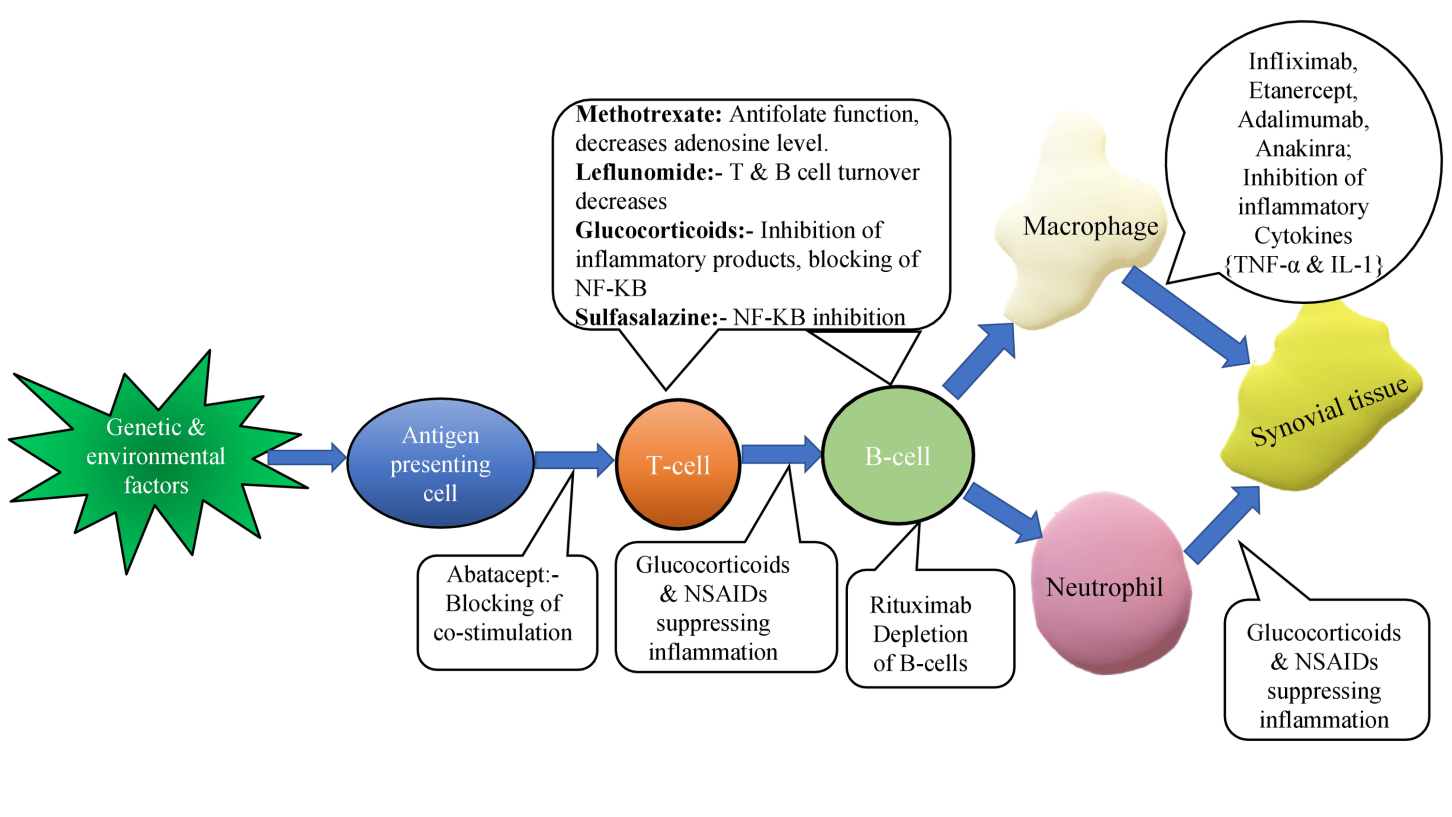
Current strategies towards management of RA. NF-KB: nuclear factor kappa B; TNF-α: tumor necrosis factor-alpha; IL-1: interleukin-1; NSAIDs: non-steroidal anti-inflammatory drugs; RA: rheumatoid arthritis. The image was illustrated by the author Sarika Patil.

The treatment landscape for RA has significantly evolved since the late 20th century. Advances in understanding its pathophysiology have led to treatment strategies that target immune-inflammatory processes, utilizing biologic and targeted synthetic DMARDs. Alongside, treat-to-target recommendations have been developed to help achieve and maintain therapeutic targets quickly, preventing structural, functional, and overall disability. These refinements in RA treatment have been integrated into guidelines from the European Alliance of Associations for Rheumatology (EULAR) and the American College of Rheumatology (ACR). These guidelines outline a treatment pathway that includes the early use of conventional synthetic (cs) DMARDs with glucocorticoids, followed by sequential treatment strategies if the therapeutic target is not reached [16,17]. Step 1 classifies RA patients into high disease activity or moderate-to-high disease activity states. High disease activity is defined as having more than six tender joints and a high-sensitivity C-reactive protein (hsCRP) level over 3.01 mg/L. Moderate-to-high disease activity is defined as having three to six tender joints and an hsCRP level over 2.02 mg/L. Patients in these states should be treated with csDMARDs like methotrexate, and combination therapy is recommended for those at higher risk compared to monotherapy. For patients who do not respond to or cannot tolerate conventional systemic agents, the next step is treatment with biologic DMARDs (bDMARDs). Recently, next-generation DMARDs called targeted synthetic DMARDs (tsDMARDs) have been developed for patients who fail to respond to bDMARDs [18]. The general treatment strategy for the management of RA is depicted in Figure 1.

Biologic therapies in rheumatoid arthritis

The standard of care in the management of RA has significantly advanced over time, aligning with the expanding comprehension of the pathophysiological mechanisms contributing to the development of this debilitating condition. Over the past two decades, significant strides have been made with the development and introduction of biologic drugs designed to target the underlying processes of RA. These novel medications have proven highly effective in mitigating symptoms and slowing disease progression, resulting in improved patient outcomes. Biologic drugs have not only established a treat-to-target strategy in RA therapy but have also paved the way for modern management approaches that emphasize disease control and preventing disability. These advances have enabled healthcare professionals to optimize patient care, aiming to mitigate or even halt RA-related impairments. However, it is important to recognize that biologic drugs are not without potential drawbacks. Adverse effects are a genuine concern for both patients and healthcare providers. Therefore, assessing and monitoring potential risks and adverse events is crucial, requiring careful consideration and evaluation by both the patient and their physician to balance therapeutic benefits with potential harm. It is imperative for researchers, clinicians, and pharmaceutical companies to continue collaborating to advance the understanding of RA's pathogenesis and refine treatment approaches. This collaborative effort aims to further optimize the efficacy and safety of existing biologic drugs while exploring new therapeutic avenues. Ultimately, this ongoing pursuit of knowledge and innovation holds the promise of transforming the lives of individuals living with RA, offering them a brighter and more hopeful future [19]. Biologic drug innovations have led to the establishment of bioregulators that work with immune system functions. Specialists have extensively explored various agents to block specific processes and cytokines contributing to immune system dysfunction in RA, including adalimumab, abatacept, certolizumab pegol, anakinra, golimumab, tocilizumab, etanercept, infliximab, rituximab, sarilumab, baricitinib, and more recently, upadacitinib. These biologics are part of standard disease management for RA patients but have also shown efficacy in other autoimmune diseases. The introduction of these new biologics has increased the capability to diagnose and treat RA. They have revolutionized treatment strategies involving disease-modifying and anti-rheumatic drugs, establishing a comprehensive and holistic approach to managing RA and other autoimmune diseases. These groundbreaking bioregulators have opened new possibilities in medicine, enhancing patient care and improving quality of life [20].

Mechanism of action

RA is a chronic autoimmune disorder characterized by inflammation. Previous treatments focused on reducing inflammation and relieving RA symptoms, but a groundbreaking new approach aims to prevent disease progression and improve quality of life by halting immune system malfunctions. This method involves developing biologics derived from recombinant DNA technology, targeting key molecules responsible for the disease. These biologics, with their low toxicity and high efficacy, have emerged as powerful tools in RA treatment, offering unparalleled advantages and targeting cytokines and T cell subsets. These innovative biological agents have shown exceptional therapeutic benefits, although they come with distinctive adverse effects. RA is a complex disorder with an unknown origin, involving excessive inflammation, aberrant immune responses, and synovial pannus formation [21-23]. The synovium of affected joints undergoes hyperemia and proliferative pannus growth, leading to the secretion of matrix metalloproteinases (MMPs). MMPs play a crucial role in cartilage destruction and synovitis development in the joints, causing joint swelling, pain, and bone deformities. As the matrix degrades, joint damage progresses, leading to disability and, in severe cases, loss of daily function. Previously, therapeutic approaches focused on pharmacological interventions to reduce inflammation and alleviate symptoms. However, a novel strategy has emerged, emphasizing immune system modulation to impede disease progression and enhance quality of life. By focusing on immune functionality, this approach aims to manage RA and prevent further complications from this chronic condition [24-26].

Approved biologic agents

Unique aspects of biologic drug technologies are the ability to efficiently block extremely specific proteins or key cells, ultimately leading to the achievement of highly targeted therapeutic consequences. Numerous innovative drug entities, such as interleukin-1 receptor antagonists, tumor necrosis factor-alpha (TNF-alpha) antibodies or soluble receptors, and B-cell blockers, significantly contribute to the ever-expanding armamentarium of biologic drugs that are currently available or expected to be developed in the immediate future. These state-of-the-art biologic agents not only provide considerable relief from debilitating symptoms in patients with RA but also have the potential to effectively delay the progression of the disease, while simultaneously limiting structural damage or even inducing complete remission in certain cases. However, it is crucial to acknowledge that all of these groundbreaking medications come with inherent risks of causing serious adverse effects and suppressing the immune system, thereby increasing the potential for the reactivation of latent tuberculosis [27-29]. The future of RA treatment is focused on strategies that precisely target the inflammatory cascade. Biologic agents, embodying cutting-edge technology, offer a vast array of possibilities for new treatments on the horizon of RA management. Unlike traditional DMARDs, biologic DMARDs specifically target immune system cells or proteins involved in the inflammatory process, effectively preventing the chronic destruction of synovial and other tissues caused by RA. Due to their targeted effects and strong immunosuppression capabilities, biologic drugs are often considered a treatment of last resort, added to the regimen when traditional DMARDs prove ineffective. Currently, Rituximab, Abatacept, and tofacitinib are available biologic drugs, with several other promising candidates pending approval. Future biologic drugs will likely include anti-interleukin 6 and other potential candidates such as osteoprotegerin ligand, anti-intercellular bond molecule 1, biologic enzymes within synovial cells, and metabolically blocking microbial agents. These advancements hold great promise for improving treatment options and outcomes for patients suffering from RA [30-32].

Emerging biologic therapies

The potential for emerging biologic therapies for RA is vast, with many innovative approaches on the horizon. In addition to traditional treatments like DMARDs and targeted therapies such as Janus kinase (JAK) inhibitors, there are several promising advancements. One area of research involves immune-tolerizing drugs, which can program the immune system to ignore specific antigens associated with RA. By desensitizing the immune system to target only harmful cells and substances, these therapies could significantly reduce inflammation and prevent tissue damage. Scientists are also developing T-regulatory drugs, which aim to increase the production of immune cells that suppress the immune response. Enhancing these regulatory cells' presence and activity may prevent the overactivation of the immune system characteristic of RA, leading to a more balanced and controlled response. Additionally, gene therapy represents an exciting frontier in RA treatment. This approach involves manipulating genetic material within cells to correct disease-causing mutations or introduce therapeutic genes addressing RA's underlying cause. By modifying cell DNA, researchers hope to provide long-lasting benefits by promoting the production of therapeutic proteins, offering a personalized approach with tremendous potential for new RA treatments [33-36]. The field of biologic therapies for RA is evolving rapidly, offering numerous exciting possibilities. Researchers are tirelessly working on innovative solutions, including immune-tolerant drugs, T-regulatory drugs, and gene therapy, aiming to revolutionize the treatment of this chronic autoimmune disease. These advancements hold the promise of not only alleviating symptoms but also halting or reversing RA progression, ultimately improving the lives of millions worldwide. Among the most promising future biologic therapies for RA are cell-based treatments, where immune cells are modified in the lab to ameliorate the disease. Induced pluripotent stem cells could regenerate damaged joints, while tissue or bone marrow-specific mesenchymal stem cells (MSCs) might deliver anti-inflammatory proteins directly into joints to resolve symptoms. Additionally, advances in gene editing technologies, such as CRISPR-Cas9, have the potential to revolutionize RA treatment by precisely modifying immune cells to target and suppress the autoimmune response. While these therapies show great promise, challenges remain in the discovery and validation of drug targets and their practical use. Despite these challenges, ongoing research and international collaborations give hope. Scientists and clinicians are optimistic about developing innovative solutions to combat RA's complexity. By unraveling the intricate mechanisms of the disease and incorporating cutting-edge technologies, researchers aim to unlock new therapeutic avenues, providing long-lasting relief and improved quality of life for RA patients [37-41]. Some biological agents are discussed below.

Rheumatoid Arthritis Synovial Fibroblasts (RASF) Triggering Molecules

Rheumatoid arthritis synovial fibroblasts (RASF) are central to the progression of RA, promoting inflammation and joint destruction. These fibroblasts are activated by a variety of molecules, which contribute to the disease's pathogenesis. By targeting these activation pathways, emerging biologic therapies aim to mitigate the impact of RASF on joint health [42].

B Cell-Activating Factor (BAFF)

BAFF is essential for B cell survival and maturation, and its overproduction is linked to autoimmune diseases like RA. Elevated BAFF levels in RA patients contribute to the autoimmune response, making BAFF a crucial target for biologic therapies. These therapies aim to reduce disease activity by inhibiting BAFF, thus preventing B cell-driven inflammation [43].

Tumor Necrosis Factor (TNF)

TNF is a key pro-inflammatory cytokine involved in RA, promoting joint inflammation and destruction. TNF inhibitors are among the most effective biologic therapies for RA, targeting this cytokine to reduce inflammation and prevent further joint damage [44].

S100 Calcium-Binding Protein A7 (S100A7, Psoriasin)

S100A7 is overexpressed in RA and plays a role in the inflammatory response by increasing the production of pro-inflammatory cytokines. Targeting S100A7 can help modulate this response, potentially reducing inflammation and joint damage in RA patients [45].

Interleukin-36α (IL-36α)

IL-36α is part of the IL-36 cytokine family and is involved in RA by stimulating pro-inflammatory mediators in synovial fibroblasts. Emerging therapies targeting IL-36α aim to reduce these inflammatory signals, providing another avenue to control RA symptoms and progression [46].

Novel targets and mechanisms of action

In recent years, there has been significant progress in understanding the pathogenesis of RA, resulting in a detailed comprehension of the cellular and molecular pathways contributing to the disease's expression and persistence. These insights have led to rigorous testing of biologically active agents in various clinical trials. Notably, some biologic agents used in clinical settings were chosen based on their specific mechanisms of action rather than a previously known target in RA [47,48]. For example, the identification of TNF as a therapeutic target emerged from positive clinical experiences with IL receptor antagonists and the effects of anti-TNF antibodies in animal models of arthritis. Additionally, other biologic agents have been developed to target specific molecules or a range of target molecules directly involved in the pathogenic process of RA [49]. The identification of targets and target molecules for RA treatment begins with extensive basic research across various scientific fields. For instance, the discovery of auto-antibodies against the triple-helix collagen structure was a pivotal breakthrough, revolutionizing the understanding of RA. This discovery led to the development of sophisticated serum tests for accurate diagnosis and disease severity prediction and revealed collagen's role in T cell activation long before its fundamental role in T cell function was understood. Despite gaps in knowledge, pharmaceutical advancements like the advent of abatacept have shown remarkable efficacy in managing this complex autoimmune disorder. Researchers have also focused on identifying key signaling molecules, co-stimulatory factors, and adhesion molecules with heightened activation or expression levels in RA patients and animal models. Significant progress has been made in disrupting disease progression, particularly through targeting dysregulated cellular adhesion processes. Advances have included antibodies designed to interfere with α4 and β1 integrins and to disrupt VLA-1/VCAM-1 interactions, which are crucial for inflammation and cell motility. These findings highlight the intricate and multifaceted nature of RA, as well as numerous strategies for manipulating and counteracting the disease. Researchers remain dedicated to unraveling the underlying mechanisms to develop novel therapeutic interventions, aiming to relieve the burdens endured by those with RA [50].

Clinical trials and research findings

In clinical trials, tofacitinib, a Janus kinase (JAK) inhibitor, has shown effectiveness, though it comes with a higher risk of adverse events like infection. Upadacitinib is under investigation in phase 3 trials, and peficitinib, another JAK inhibitor, has also proven effective when combined with conventional DMARDs. Tofacitinib and baricitinib are currently marketed for treating RA. For patients not responding to their current treatments, switching to a different JAK inhibitor or targeted synthetic DMARD can be considered [51,52]. JAK therapy is another novel intervention. Although the breakdown or malfunction of immune tissues in RA is still being studied, many cytokines mediating intracellular processes play a crucial role in RA's pathogenesis. JAK kinases mediate the phosphorylation of signal transducer and activator of transcription (STAT) in the JAK-STAT pathway. TYK2 and JAK1 kinases are particularly effective in initiating this process, playing critical roles in the immune response. JAK inhibitors target tyrosine kinase, blocking multiple cytokine signals in the cell. Blocking TYK2 can potentially limit proinflammatory messaging, but it causes significant toxicity in humans. Selective inhibitors targeting specific JAK kinases are being developed to reduce side effects in RA treatment. These selective inhibitors may be more likely to succeed than broad kinase inhibition [53,54].

Challenges and future directions

The use of biologic agents to target multiple signal transduction pathways highlights the complexity of RA pathogenesis and the potential for interference at various levels of disease induction. As experience with biologic agents increases in RA, animal models, and other chronic diseases, we are becoming more sophisticated in their application. However, the long-term safety of biologics must be carefully evaluated in future studies. Blocking cytokines with biologics can lead to latent tuberculosis (TB) infection and reactivation of herpes simplex virus (HSV). Cytokines play a role in apoptosis and muscle wasting, which may explain why patients treated with TNF-alpha inhibitors do not gain weight. This also has implications for treating cachexia in patients receiving anti-IFN-alpha for chronic hepatitis [55-57]. Cytokine-targeted therapy is highly effective for treating RA. TNF-alpha blocking agents have shown superior efficiency, although predicting response factors remains uncertain. The presence of anti-hepatitis B antibodies (AHBs) may indicate a poor response. While serious side effects like infections, autoimmune diseases, lymphoma, and congestive heart failure are relatively rare, further studies are needed to assess the safety and efficacy of TNF-alpha blockers. The high cost of biological therapy limits its widespread use. However, a recent meta-analysis revealed that biologics are cost-effective for patients with longstanding RA who have not responded to conventional DMARDs. As our understanding of RA's molecular and cellular events advances, we can expect significant breakthroughs in developing targeted and personalized therapies for this complex disease [58,59].

Efficacy and safety concerns

The potential of emerging biologic therapies for RA raises both short- and long-term safety concerns due to limited experience with these treatments and the extent to which they manipulate the host immune system. To address safety, it is crucial to understand the role of targeted cytokines within the immune system, their redundancy, and their role in protecting against infections, including bacterial, fungal, and parasitic. Evaluating safety data in target populations, pharmacokinetics, and risk versus benefit is also essential. In the natural immune system, cytokine production involves redundancy and antagonistic factors, playing a broad and protective role against various infections. Understanding this role is critical, as the relative redundancy of cytokine production can only be ethically and practically assessed through experience. A major concern with any agent manipulating the immune system, particularly in chronic autoimmune diseases like RA, is the potential for long-term adverse effects [60]. Non-selective agents such as cyclophosphamide or chlorambucil, while highly effective at controlling RA, pose risks for short- and long-term adverse effects like infection and malignancy. Despite these risks, these agents are considered effective, non-selective, simple, and inexpensive. The potential of emerging biologic therapies for RA raises both short- and long-term safety concerns due to limited experience with these treatments and the extent to which they manipulate the host immune system. To address safety, it is crucial to understand the role of targeted cytokines within the immune system, their redundancy, and their role in protecting against infections, including bacterial, fungal, and parasitic. Evaluating safety data in target populations, pharmacokinetics, and risk versus benefit is also essential [61,62].

Personalized medicine approaches

Personalized medicine for RA is gaining significant interest, aiming for precision in treatment. Different approaches are exploring ways to define opportunities for personalized RA management, with promising results seen in combining numerous biomarkers within an ACPA+ context. This involves integrating clinical, genetic, epigenetic, protein, immunological, and histopathological features to use proteins as quantitative biomarkers for RA initiation, prognosis, and drug response. Data mining of molecular layers promises to enhance the identification of patient sub-types at an individual level. These combinations can be further improved by examining disease-relevant cellular changes contributing to RA sub-types through multi-omics integration [63-65]. As researchers study these molecular subtypes and explore newly characterized pathways or potential intervention targets, there are important opportunities for developing poly-ADP ribose polymerase (PARP) inhibitors for specific RA sub-types. Such findings might be beneficial alongside current paracrine biologic therapies targeting cellular heterogeneity. It remains uncertain whether these benefits come from simple physical drug removal or the opportunity to broaden the efficacy of KLD, a blend of enzymes that includes kinase, ligase, and DpnI-part of the DpnI/DpnII pneumococcal system. This system defends against foreign attacks while allowing genetic exchange. Current biologic research focuses on treating T cells, with cellular subsets needing to differentiate the effects of cellular heterogeneity and understand effector and regulatory functions. Highlighting these personalized therapy approaches' challenges will be crucial moving forward [66-71].

Table 1 highlights the key differences and advancements in RA treatment, showcasing how novel biological drugs offer more targeted and potentially effective options with fewer side effects, albeit at a higher cost [72,73].

**Table 1:** Comparative study between conventional and novel treatments and biological drugs. TNF-α: tumor necrosis factor-alpha; IL-6: interleukin-6.

Sr. No.	Aspect	Conventional/old treatments	Novel biological drugs
1.	Mechanism of action	Non-selective immunosuppression	Target specific molecules/pathways
2.	Examples	Methotrexate, hydroxychloroquine, sulfasalazine	TNF-α inhibitors (e.g., adalimumab), IL-6 inhibitors (e.g., tocilizumab)
3.	Efficacy	Effective in controlling symptoms	Often more effective in achieving remission
4.	Side effects	Broader range of side effects (e.g., liver toxicity, bone marrow suppression)	More targeted, potentially fewer side effects
5.	Administration	Oral or injectable	Usually injectable
6.	Cost	Generally lower cost	Higher cost due to biotechnological production
7.	Usage	First-line treatment	Used when conventional treatments fail or for more severe cases

## Conclusions

RA is a common autoimmune disease marked by cartilage and bone loss initiated by the activation and proliferation of synovial fibroblasts. Current RA treatments often involve broad immunosuppression protocols. The search for more specific RA treatments without the side effects associated with broad immunosuppression has led to new concepts regarding RA pathogenesis, involving novel treatments and biologic drugs with new mechanisms. In the present review, we discuss the potential of emerging RA biologic therapy centered on RASF (rheumatoid arthritis synovial fibroblasts) triggering molecules, B cell activating factor (BAFF), a protein of the tumor necrosis factor (TNF) family, and alarmins S100 calcium-binding protein A7 (S100A7, also known as psoriasin) and IL-36α in the pathogenesis of RA. As these alarmins could be involved in the pathogenesis of inflammatory joint diseases, they might present therapeutic targets for drugs in the treatment of such diseases. There are currently no known endogenous complete gene silencing mechanisms. Products of S100A7 and other alarmin family genes are involved in RA pathogenesis apart from their ligand-receptor process, and paclitaxel, a promoter of the nuclear translocation of S100A7, might be an abrogation strategy for gene therapy. Similarly, S100A7 polymorphisms regulating gene expression might affect RA susceptibility, severity, and risk of early onset of arthritis, potentially as well as the therapeutic response to either glucocorticoid or adrenocorticotrophic hormone. While the involvement of RASF-triggering molecules BAFF, S100A7, and IL-36α in the pathogenesis of RA is intriguing, they are still potential targets, and their potential as RA treatments needs to be investigated further.
